# Memory for action: from cognitive models to clinical evaluation

**DOI:** 10.3402/snp.v5.26091

**Published:** 2015-11-05

**Authors:** Mathieu Hainselin

**Affiliations:** Université de Picardie Jules Verne, Amiens, France

Memory mostly concerns actions we perform, imagine, or observe: what we ate for lunch, where we walked to, who we interacted with. The worldliest common question asked while coming home is ‘What did you do today?’, which concerns our memory for action (Hainselin, Quinette, & Eustache, 2013; Madan & Singhal, 2012). Most of the literature about memory excludes actions, but some researchers studied it in the early 1980s. After two decades with little research concerning memory for action, this topic is now trending again. Recently, psychology, medicine, and neuroscience have developed new, different methods to explore embodied cognition, enactment effect, or mirror neurons, through lifespan for healthy participants or patients. But the specificity of action in memory needs further investigation to be better understood. This *Socioaffective Neuroscience & Psychology* ‘Memory and Action’ special issue gives us a great opportunity to bring together different perspectives for research on this crucial topic and to go beyond those questions.

From a theoretical point of view, the Declerck paper (2015) gives some keys on ‘how we remember what we can do’ and will bring many discussions in the future on the respective role of action, awareness, and cognition to perceive and remember actions.

Tallet, Albaret, and Rivière (2015) focused their paper on child development. They teach us how the maturity of the motor system probably relies on persistence and flexibility, by reanalyzing the well-known procedural learning and C-not-B task.

As far as children, adults and old people memory can benefit from action. Silva, Pinho, Souchay, and Moulin (2015) highlighted the advantages and limitations of the enactment effect (improvement in memory when participants perform actions while learning compared with merely reading).

Finally, those articles, (Declerck, 2015; Silva, Pinho, Souchay, & Moulin, 2015; Tallet, Albaret, & Rivière, 2015), by helping us to understand memory for action and its cognitive models, should lead researchers and clinicians from different fields to develop new therapeutic strategies, using actions in the environment, the enactment effect, and procedural learning to help children, adults, and the elderly, with or without memory problems (Hainselin et al., 2014).

*Mathieu Hainselin, PhD* Associate Professor Université de Picardie Jules Verne Amiens, France

## References

[CIT0006] Declerck G (2015). How we remember what we can do. Socioaffective Neuroscience & Psychology.

[CIT0001] Hainselin M, Quinette P, Eustache F (2013). Qu'est-ce que la mémoire de l'action? Revue théorique et perspectives. Revue de Neuropsychologie.

[CIT0002] Hainselin M, Quinette P, Juskenaite A, Desgranges B, Martinaud O, de La Sayette V (2014). Just do it! How performing an action enhances remembering in transient global amnesia. Cortex.

[CIT0003] Madan C. R, Singhal A (2012). Using actions to enhance memory: Effects of enactment, gestures, and exercise on human memory. Frontiers in Psychology.

[CIT0004] Silva A. R, Pinho M. S, Souchay C, Moulin C. J. A (2015). Evaluating the subject-performed task effect in healthy older adults: Relationship with neuropsychological tests. Socioaffective Neuroscience & Psychology.

[CIT0005] Tallet J, Albaret J.-M, Rivière J (2015). The role of motor memory in action selection and procedural learning: Insights from children with typical and atypical development. Socioaffective Neuroscience & Psychology.

